# Positivity Ratio and Well-Being Among Teachers. The Mediating Role of Work Engagement

**DOI:** 10.3389/fpsyg.2020.01608

**Published:** 2020-07-22

**Authors:** Petruta P. Rusu, Aurora A. Colomeischi

**Affiliations:** Department of Educational Sciences, University “Stefan cel Mare” of Suceava, Suceava, Romania

**Keywords:** positive emotions, negative emotions, positivity ratio, work engagement, teachers’ well-being

## Abstract

Previous studies indicated that the balance of positive to negative affect (i.e., positivity ratio) is associated with subjective well-being and flourishing in the general population. Moreover, a positivity ratio of 2.9 is considered a critical value discriminating between flourishing and non-flourishing individuals. To date, however, there is limited research on the positivity ratio on samples of teachers. The present study aimed to investigate whether the positivity ratio affects work engagement and well-being among teachers. Based on the broaden-and-build theory (Fredrickson, 2001) and work engagement model (Bakker and Demerouti, 2007), we predicted that positivity ratio (the ratio between positive and negative emotions) experienced by teachers would increase their work engagement, which in turn would positively affect their well-being. A sample of 1,335 teachers (762 women and 573 men) from Romania participated in the study. Results revealed that work engagement mediated the relationship between positivity ratio and well-being. Specifically, teachers with a higher ratio of positive to negative emotions reported more engagement (dedication, absorption, and vigor) and in consequence higher levels of subjective well-being (autonomy, environmental mastery, personal growth, self-acceptance, positive relations with others and purpose in life). Also, when investigating the positivity ratio according to participants’ well-being, we found a mean of positivity ratio of 2.84 for the group of teachers with high levels of well-being, validating the proposed critical positivity ratio of 2.9. These findings support the importance of addressing positive emotions and positivity ratio in prevention and intervention programs with teachers.

## Introduction

Teachers are emotional workers (Yin, 2015). Teachers’ emotions have been found to influence their performance, self-efficacy, job satisfaction, burnout, and instructional effectiveness (classroom management, teacher support for students, student-centered approaches to teaching, and cognitive and motivational stimulation provided to students) (Frenzel, 2014; Taxer and Frenzel, 2015; Lavy and Eshet, 2018; Chen, 2019a, b). Thus far, most of the existing studies focused on teachers’ negative emotions such as depression or anxiety and their associations with job burnout and occupational stress (Desouky and Allam, 2017; Capone et al., 2019). Although empirical research on teachers’ emotions increased in the last decade, there is still limited research considering teachers’ positive emotions and how positive emotions affect individual and organizational outcomes. This is surprising since teachers experience at the same time negative emotions (such as anger, anxiety, shame, and boredom) and positive emotions (such as enjoyment, pride, love and caring) (Frenzel, 2014; Buriæ et al., 2018, 2019; Lee and van Vlack, 2018). According to the broaden-and-build theory (Fredrickson, 2001), experiencing positive emotions prompt individuals to be more engaged in activities and increase their psychological well-being. Similarly, the happy-productive-worker theory conceptualizes positive emotions as antecedents of work engagement and satisfaction (see for review García-Buades et al., 2019). Organizational studies indicated that positive emotions have been positively associated with self-efficacy, hope, optimism, job satisfaction, and flow at work (Siu et al., 2015; Zito et al., 2019). Another line of research considered that an important predictor of well-being and flourishing is the balance between positive and negative affect (i.e., positivity ratio) (Fredrickson and Losada, 2005). Positivity ratio was positively related to life satisfaction, self-control, self-esteem, and optimism (Shrira et al., 2016; Orkibi and Ronen, 2017). However, only a few studies considered positivity ratio in teachers, and to our knowledge, there is no empirical study in educational context investigating the associations of positivity ratio with work engagement and well-being. Moreover, most of the existing studies involving teachers’ emotions have been conducted on samples of teachers from the United States and Western Europe, and the generalization of the findings to teachers from Eastern Europe may be limited. The present study aims to address these gaps by investigating whether teachers’ positivity ratio is associated with their well-being through the mediating role of work engagement. Based on the aforementioned theories and research, we suggest not only that positive emotions are important but also that the affective balance between positive and negative emotions would be associated with teachers’ engagement and well-being. Given the limited number of studies on teachers from Eastern Europe, the current study focused on a sample of teachers from Romania.

### Benefits of Teachers’ Positive Emotions

Positive emotions transform both individuals and organizations, leading to growth and development (Fredrickson, 2000). The broaden-and-build theory (Fredrickson, 2001) assumes that positive emotions broaden the array of thoughts and actions (e.g., to explore, to be creative, and to savor life experiences), which in consequence build personal resources (e.g., psychological well-being and resilience). Studies based on the broaden-and-build theory indicated that positive emotions at work contribute to positive outcomes, including enduring personal resources (such as work engagement, positive beliefs, creativity, and effective coping strategies), social outcomes (such as good relationships at work and cooperation), and job performance (see, for review, Diener et al., 2020). In general, positive emotions have been positively associated with work well-being, self-efficacy, hope, optimism, adaptive coping, resilience, job satisfaction, emotional intelligence, creativity, and flow at work (Parke et al., 2015; Siu et al., 2015; Gloria and Steinhardt, 2016; Zito et al., 2019). In contrast, existing studies reported negative relationships between positive emotions and stress symptoms, turnover intentions, maladaptive coping, depression, and anxiety (Siu et al., 2015; Gloria and Steinhardt, 2016). Positive emotions also trigger upward spirals, as their positive consequences predict future increases in positive emotions and lead further to well-being (Fredrickson and Joiner, 2002, 2018).

In the context of teaching profession, studies indicated that teachers’ positive emotions have been positively related to their self-efficacy, work engagement, performance, job satisfaction, life satisfaction, enthusiasm, and positive student behaviors, while negative emotions have been associated with less work engagement, lower levels of self-efficacy, and lower performance (Brígido et al., 2013; Buriæ and Macuka, 2018; Chen, 2019b; Buriæ and Moè, 2020). Specifically, positive emotions of joy and love in teaching have been positively associated with performance, teachers’ sense of self-efficacy, student-centered approaches to teaching, and student focus (Chen, 2019a, b). Also, a recent longitudinal study revealed that positive affective experiences have an important role in shaping teachers’ self-efficacy and job satisfaction, which in turn were positively related to teachers’ enthusiasm (Buriæ and Moè, 2020). The spiral effects of emotions have been supported in a daily diary conducted on a sample of teachers, which showed that teachers’ daily positive emotions trigger an upward spiral leading to job satisfaction, while negative emotions prompt a downward spiral leading to burnout (Lavy and Eshet, 2018). Existing research considering other emotional aspects of teachers’ lives showed that teachers’ adaptive emotion regulation strategies (such as reappraisal) and emotional intelligence were positively associated with long-term well-being, job satisfaction, and expression of naturally felt emotions, while emotional job demands of teaching affected teachers’ well-being by determining emotional exhaustion (Yin, 2015; Yin et al., 2016, 2018, 2019).

### Positivity Ratio

Positivity ratio, the ratio of positive to negative affect, was conceptualized as a key predictor of well-being and flourishing (Fredrickson and Losada, 2005). Moreover, research suggests that the critical value of the positivity ratio distinguishing between flourishing and non-flourishing individuals is 2.9:1 (Fredrickson and Losada, 2005).

This specific value of positivity ratio and the formula for calculating it have been criticized later on (Brown et al., 2013). Moreover, a recent study indicated a curvilinear relationship between positivity ratio and exhaustion, suggesting that after exceeding a specific value (i.e., 2.0), the positivity ratio may lead to negative outcomes, such as work exhaustion (Basińska and Gruszczyńska, 2017). Despite the criticisms and disapprovals regarding the existence of a specific value for the positivity ratio for distinguishing between “flourishing” and “languishing” individuals, Brown et al. (2013) did not criticize the construct on its own and they agreed that positivity ratio might be associated with positive outcomes. Thus far, research found positive associations of positivity ratio with emotional intelligence, life satisfaction, optimism, self-esteem, and self-control (Shrira et al., 2016; Orkibi and Ronen, 2017; Moroń, 2018) and negative associations with job burnout (Basińska and Gruszczyńska, 2017).

A few empirical studies investigated the ratio between positive and negative emotions in an educational context. Specifically, teachers’ hedonic balance (computed as the difference between positive and negative emotions) was positively associated with teachers’ self-efficacy, job satisfaction, and student-related positive emotions (Buonomo et al., 2019, 2020). Research supporting the broaden-and-build theory among teachers also revealed that positive emotions can reduce the negative effects of negative emotions (i.e., undoing effect) (Gloria et al., 2013; Buonomo et al., 2019). At the same time, studies investigating both teachers’ positive and negative emotions revealed that in general low levels of positive emotions reported by teachers (such as enjoyment and pride) and high levels of negative emotions (such as anger and anxiety) correspond to higher levels of emotional exhaustion, teacher burnout, and emotional labor (Keller et al., 2014; Khajavy et al., 2017; Wang et al., 2019). Therefore, we suggest that the balance of positive to negative emotions should be considered in studies investigating teachers’ well-being. The present study will investigate the relationships between positivity ratio, work engagement, and psychological well-being among Romanian teachers.

### Emotions, Work Engagement, and Well-Being

Based on the job demands–resources model (JD-R Model, Bakker and Demerouti, 2007), higher levels of employee well-being are determined by job resources through work engagement, while lower levels of well-being are predicted by job demands through burnout. Work engagement was defined as a positive attitude toward work characterized by vigor (high levels of energy and perseverance), dedication (work involvement, enthusiasm, and inspiration), and absorption (work immersion and concentration) (Schaufeli et al., 2002).

Studies testing the JD-R model showed that personal resources (self-esteem, optimism, self-efficacy, and active coping) have been positively related to work engagement and psychological well-being and negatively associated with exhaustion (Xanthopoulou et al., 2007; Lee, 2019). Positive emotions play also an important role in work engagement. Fredrickson (2000) suggested that the items used by Gallup for measuring employees’ engagement target indirectly positive emotions and that the positive influence of engagement on organizational outcomes derives from positive emotions. A review including cross-sectional, longitudinal, and experimental studies indicated that positive emotions affect work life as they are related to better work quality, higher job performance, cooperation, reduced conflict with colleagues, prosocial organizational behavior, and better income (Lyubomirsky et al., 2005). In general, work engagement was positively related to positive emotions and negatively associated to negative emotions and emotional exhaustion (Sonnentag et al., 2008; Malinowski and Lim, 2015; Dicke et al., 2018; Bakker et al., 2019; Moreira-Fontán et al., 2019).

Studies conducted on teachers support the beneficial effects of positive emotions on work performance, teaching self-efficacy, mental health, and job satisfaction (Taxer and Frenzel, 2015; Lavy and Eshet, 2018). Buriæ and Macuka (2018) found that teachers’ positive emotions of joy, love, and pride have been related to higher levels of work engagement 6 months later, while negative emotions of anger, fatigue, and hopelessness were negatively related to engagement. In contrast, another study showed that emotional exhaustion was negatively related to teachers’ work engagement and job satisfaction (Han et al., 2019).

Regarding the relationship between work engagement and well-being, existing research found positive associations. Higher levels of cognitive, emotional, and physical engagement have been positively related to well-being and personal accomplishment (Shuck and Reio, 2014). Vigor, dedication, and absorption have been positively associated with job satisfaction (Yan et al., 2019). Recent empirical studies also found positive relationships between work engagement and job satisfaction among teachers (Perera et al., 2018; Han et al., 2019). Most of the existing studies testing the JD-R model conceptualized well-being through job satisfaction, work burnout, and emotional exhaustion (Mencl et al., 2016; Khajavy et al., 2017; Ferreira et al., 2019; Lesener et al., 2019), and few studies investigated psychological well-being. The present study will investigate whether teachers’ emotions are linked to work engagement and psychological well-being.

### Hypotheses

According to the broaden-and-build theory (Fredrickson, 2001) and JD-R model (Bakker and Demerouti, 2007) and based on existing studies showing significant associations of positive and negative emotions with work engagement (Sonnentag et al., 2008; Malinowski and Lim, 2015; Buriæ and Macuka, 2018) and psychological well-being (Lavy and Eshet, 2018), we first assumed that positive emotions and the positivity ratio will be positively associated with teachers’ work engagement and psychological well-being. Second, considering the positive associations of work engagement with well-being and flourishing (Robledo et al., 2019; Tesi et al., 2019), we predicted that teachers’ work engagement will be positively related to psychological well-being. Third, as our main hypothesis, we predicted that teachers’ work engagement will mediate the association of emotions and positivity ratio with psychological well-being. This hypothesis is derived from the JD-R model (Bakker and Demerouti, 2007) and studies supporting this model (see for review Lesener et al., 2019), assuming that personal resources are related to positive individual and organizational outcomes. In order to test these hypotheses, we tested two separate models, one considering positive and negative emotions separately as independent variables and the second one considering the positivity ratio as an independent variable.

## Method

### Sample

The sample consisted of 1,335 teachers (762 women and 573 men). Participants had a mean age of 39.19 (*SD* = 10.55, range = 18–68). On average, teachers had a work experience of 15.04 years (*SD* = 11.07, range = 0–48 years). Participating teachers worked in all school levels: preschool (16.4%), primary school (39.6%), middle school (30%), and high school (14.1%). Approximately half of the teachers worked in an urban area (53.3%) and half of them in a rural area (46.7%). Regarding their marital status, 74.4% were married, 21.4% were not married, 2.7% were divorced, and 1.4% were widowed.

### Procedure

Participating teachers in the present study were recruited by students attending an educational science program from a public Romanian university. Students were instructed to distribute the questionnaires to teachers. The Institutional Review Board of the Romanian University approved the study. All participants signed informed consent. Teachers were not reimbursed for participation in this study. Students received course credits for data collection.

### Measures

Teachers completed demographic information regarding age, gender, education, work experience, marital status, and school level where they teach.

#### Emotions

The Positive Affect and Negative Affect Scale (PANAS, Watson et al., 1988) was used to assess positive and negative emotions. PANAS is a 20-item questionnaire that measures positive emotions (e.g., enthusiastic, proud, and inspired) and negative emotions (e.g., distressed, upset, and guilty) by asking participants to rate the frequency with which they experience various emotions on a 5-point Likert scale ranging from 1 (very slightly or not at all) to 5 (extremely). Internal consistency in this study was α = 0.78 for positive affect and α = 0.80 for negative affect.

#### Work Engagement

The Utrecht Work Engagement Scale (UWES, Seppälä et al., 2009) was used to measure teachers’ engagement at work. UWES consists of 17 items grouped into three factors: vigor (e.g., *At my work, I always persevere, even when things do not go well*), dedication (e.g., *I find the work I do full of meaning and purpose*), and absorption (e.g., *When I am working, I forget everything else around me*). Participants reported their level of agreement with each item on a 7-point Likert scale ranging from 0 (never) to 6 (always/every day). In our study, Cronbach’s alpha was α = 0.78 for each subscale.

#### Well-Being

The Psychological Well-being Scale (Ryff, 1989) was used to measure teachers’ well-being. The scale consists of 84 items which measure the six underlying dimensions of well-being: autonomy (e.g., *My decisions are not usually influenced by what everyone else is doing*), personal growth (e.g., *I have the sense that I have developed a lot as a person over time*), self-acceptance (e.g., *In many ways, I feel disappointed about my achievements in life*), positive relations with others (e.g., *People would describe me as a giving person, willing to share my time with others*), environmental mastery (e.g., *I do not fit very well with the people and the community around me*), and purpose in life (e.g., *I enjoy making plans for the future and working to make them a reality*). Items are rated on a 6-point Likert scale from 1 (completely disagree) to 6 (completely agree). In the present study, Cronbach’s alpha was α = 0.77 for autonomy, α = 0.76 for personal growth, and α = 0.75 for self-acceptance, positive relations, environmental mastery, and purpose in life.

### Analytic Strategy

**FIGURE 1:**
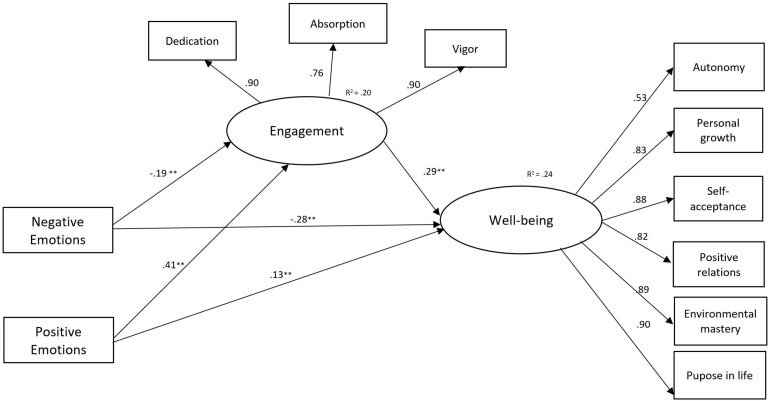
Standardized coefficients for the first mediation model, including positive emotions, negative emotions, engagement and well-being. Note: *n* = 1335, **p < 0.01 (two-tailed).

**FIGURE 2:**
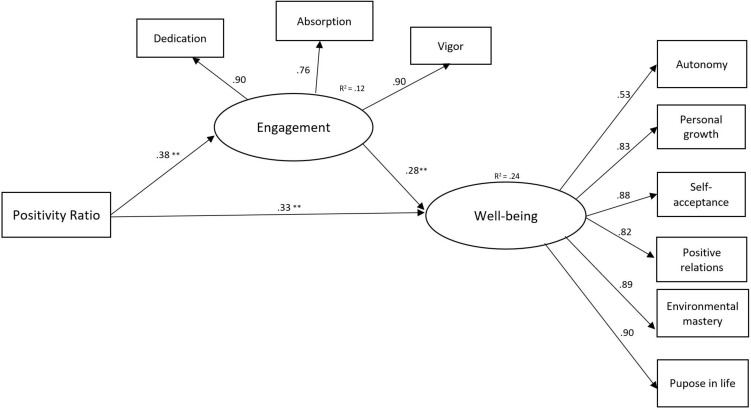
Standardized coefficients for the second mediation model, including positivity ratio, engagement and well-being. Note: *n* = 1335, **p < 0.01 (two-tailed).

We used SPSS 22 for descriptive statistics, correlations, and *t*-tests. In order to estimate the structural model, we used Mplus 7.11 (Muthén and Muthén, 1998). The following common fit indices were considered: comparative fit index (CFI), Tucker–Lewis index (TLI), standardized root mean square residual (SRMR), and root mean square residual of approximation (RMSEA) (Schermelleh-Engel et al., 2003). We used full information maximum likelihood estimator and bootstrap option in Mplus to compute model parameters and standard errors. In testing the mediation models, positive and negative emotions and positivity ratio were considered as manifest variables, while engagement and well-being were considered as latent variables (Figures 1, 2).

## Results

### Descriptive Statistics

Means, standard deviations, and the results of the *t*-test for independent samples across gender for each of the study variables are presented in Table 1. Overall, teachers reported relatively moderate levels of positive emotions and low levels of negative emotions. In terms of engagement, participants reported moderate levels of dedication, moderate levels of absorption, and high levels of vigor. In regard to well-being, participants indicated moderate levels of autonomy, personal growth, self-acceptance, positive relations, environmental mastery, and purpose in life. Results also indicated that women reported higher levels of dedication, absorption, autonomy, personal growth, purpose in life, and higher total scores for engagement and well-being than men. In addition, men reported higher levels of negative affect than did women.

**TABLE 1:** Descriptive statistics and independent sample *t*-test for path model variables.

Variable	Mean	SD	Mean difference	*t*	Cohen’s *d*
Positive affect					
Women	35.94	6.36	0.27	0.77	0.04
Men	36.21	6.42			
Negative affect					
Women	16.07	6.36	0.75*	2.12	0.11
Men	16.83	6.47			
Positivity ratio					
Women	2.53	0.91	–0.08	–1.63	0.09
Men	2.44	0.93			
Dedication					
Women	25.43	3.99	−0.80**	–3.68	0.20
Men	24.62	3.87			
Absorption					
Women	27.80	5.15	−1.33**	–4.58	0.25
Men	26.47	5.37			
Vigor					
Women	28.73	4.67	–0.35	–1.41	0.07
Men	28.37	4.46			
Engagement					
Women	81.96	12.67	−2.49**	–3.61	0.19
Men	79.47	12.23			
Autonomy					
Women	55.99	7.42	−1.12**	–2.70	0.14
Men	54.87	7.63			
Personal growth					
Women	63.70	9.28	−1.77**	–3.49	0.19
Men	61.93	9.08			
Self-acceptance					
Women	62.87	10.11	–1.07	–1.94	0.10
Men	61.80	9.81			
Positive relations					
Women	63.86	10.55	–0.95	–1.62	0.09
Men	62.90	10.77			
Environmental mastery					
Women	62.98	9.66	–0.32	–0.59	0.03
Men	62.66	9.90			
Purpose in life					
Women	64.33	9.78	−1.55**	–2.86	0.15
Men	62.77	9.88			
Well-being					
Women	373.75	47.96	−6.81*	–2.53	0.13
Men	366.94	49.43			

*n* = 762 women and 573 men, *df* = 1333. *p < 0.05 (one-tailed). **p < 0.01 (two-tailed).

The intercorrelations between the study variables are presented in Table 2. Results show significant positive correlations of positivity ratio with engagement subscales (dedication, absorption, and vigor) and well-being subscales (autonomy, personal growth, self-acceptance, positive relations, environmental mastery, and purpose in life). Moreover, the three engagement scales were significantly positively correlated with well-being subscales.

**TABLE 2:** Correlations between the study variables.

Variables	1	2	3	4	5	6	7	8	9	10	11	12	13
(1) Positive Affect	–												
(2) Negative Affect	0.00	–											
(3) Positivity Ratio	0.46**	-0.82**	–										
(4) Dedication	0.36**	-0.17**	0.32**	–									
(5) Absorption	0.29**	-0.08**	0.21**	0.70**	–								
(6) Vigor	0.39**	-0.19**	0.35**	0.81**	0.69**	–							
(7) Engagement	0.38**	-0.15**	0.31**	0.91**	0.90**	0.92**	–						
(8) Autonomy	0.12**	-0.22**	0.26**	0.20**	0.11**	0.20**	0.18**	–					
(9) Personal Growth	0.21**	-0.26**	0.33**	0.31**	0.21**	0.31**	0.30**	0.46**	–				
(10) Self Acceptance	0.20**	-0.33**	0.38**	0.31**	0.20**	0.32**	0.30**	0.48**	0.70**	–			
(11) Positive Relations	0.24**	-0.26**	0.34**	0.31**	0.20**	0.32**	0.30**	0.41**	0.70**	0.74**	–		
(12) Environmental Mastery	0.27**	-0.31**	0.40**	0.34**	0.23**	0.38**	0.35**	0.49**	0.74**	0.80**	0.74**	–	
(13). Purpose in life	0.19**	-0.27**	0.32**	0.32**	0.20**	0.32**	0.30**	0.48**	0.78**	0.81**	0.72**	0.81**	–
(14) Well-Being	0.24**	-0.33**	0.40**	0.35**	0.23**	0.37**	0.34**	0.63**	0.86**	0.90**	0.86**	0.90**	0.91**

**p < 0.01 (two-tailed). *n* = 1335 participants.

### Path Analysis Results

The first model included positive and negative emotions as independent variables, well-being as a dependent variable, and work engagement as a mediator (Figure 1). Model 1 showed a satisfactory fit: CFI = 0.98, TLI = 0.97, RMSEA = 0.05, 90% CI [0.04,0.06], SRMR = 0.02. The second model included positivity ratio as an independent variable (Figure 2). This model had also acceptable fit indices: CFI = 0.98, TLI = 0.97, RMSEA = 0.06, 90% CI [0.05,0.06], SRMR = 0.02.

### Direct Effects

In Model 1 (Figure 1), the results indicated that positive emotions had a positive effect on engagement (β = 0.41, *p* < 0.001) and well-being (β = 0.13, *p* < 0.001) while negative emotions had a negative effect on both engagement (β = −0.19, *p* < 0.001) and well-being (β = −0.28, *p* < 0.001). Moreover, engagement had a positive direct effect on well-being (β = 0.29, *p* < 0.001).

In Model 2 (Figure 2), we considered the ratio of positive to negative emotions as a predictor of engagement and well-being. The findings revealed that the positivity ratio had a significant positive direct effect on engagement (β = 0.38, *p* < 0.001) and well-being (β = 0.33, *p* < 0.001), while engagement had a significant positive direct effect on well-being (β = 0.28, *p* < 0.001).

### Indirect Mediation Effects

In Model 1, both positive emotions and negative emotions had an indirect effect on well-being through the mediating role of engagement (β = 0.12, *p* < 0.01 for positive emotions and β = −0.05, *p* < 0.01 for negative emotions).

In Model 2, the results showed that the positivity ratio had a significant indirect effect on well-being through the mediating role of engagement (β = 0.11, *p* < 0.01).

## Discussion

Being a teacher is an important role that involves generativity, as teachers influence not only students but also the whole community through the dissemination of knowledge, values, and beliefs (Branand and Nakamura, 2016). Thus, it is important to understand the antecedents of teachers’ well-being. The present study aimed to examine whether the positivity ratio is associated with teachers’ well-being directly and indirectly by affecting the engagement at work. Our study was an extension of the broaden-and-build theory of positive emotions (Fredrickson, 1998, 2001) in the educational context. Our findings showed positive associations of personal resources (positivity ratio) with work engagement and well-being, supporting the JD-R model and previous studies based on it (Bakker and Demerouti, 2007; Xanthopoulou et al., 2007; Lee, 2019).

First, the hypothesized positive association of positive emotions and positivity ratio with engagement was supported. The results indicated that positive emotions (Figure 1) and the positivity ratio (Figure 2) were significantly related to higher levels of engagement (dedication, absorption, and vigor). These findings are in line with the JD-R model and studies suggesting a positive influence of positive emotions on teachers’ work engagement (Buriæ and Macuka, 2018; De Stasio et al., 2019). Our results also indicated a negative effect of negative emotions on engagement (Figure 1). These findings are in line with studies showing that negative emotions are negatively related to work engagement (Buriæ and Macuka, 2018; Ferreira et al., 2019). By considering the ratio between positive and negative emotions, our results offer more light on the importance of teachers’ emotions on positive occupational and personal outcomes. Besides, when investigating the positivity ratio according to participants’ well-being, we found a mean of positivity ratio of 2.84 for the group of teachers with high levels of well-being. This finding confirms the proposed critical positivity ratio of 2.9 for adults with high levels of well-being (Fredrickson and Losada, 2005; Chen et al., 2017). Higher ratios of positive to negative emotions might also enhance learning and have positive effects on the teacher–student relationship (Cook et al., 2017; Sabey et al., 2019).

Also, as expected in the second hypothesis, we found engagement to be positively associated with teachers’ well-being. These results support the JD-R model and are in line with findings showing positive associations of work engagement with well-being and job satisfaction (Shuck and Reio, 2014; Perera et al., 2018; Han et al., 2019; Yan et al., 2019).

Third, our findings provided evidence for the mediation hypotheses, revealing that teachers’ engagement (dedication, absorption, and vigor) explained the effect of emotions and positivity ratio on their well-being. Our results indicate that both positive emotions and negative emotions had an indirect effect on well-being through the mediating role of engagement (Figure 1). In addition, the findings suggest that experiencing more positive than negative emotions builds teachers’ engagement, which in turn broadens their well-being (Figure 2). This study confirms the beneficial effects of positive emotions on behaviors. Our findings are consistent with prior studies finding positive associations between positive emotions and psychological well-being (Siu et al., 2015; Lavy and Eshet, 2018). As suggested by the broaden-and-build theory, positive emotions may restore other psychological resources and protect from the detrimental effect of negative emotions (Fredrickson, 2000). The bouncing-back effect of positive emotions has been confirmed in a recent study involving teachers (Buonomo et al., 2019).

### Educational Implications

Initial training of teachers should focus more on strategies to promote positive emotions and to regulate negative emotions in times of stress. The results of our study emphasize the importance of addressing both negative and positive emotions in prevention and intervention programs with teachers. Based on our findings, experiencing about three times more positive than negative emotions in daily life could help teachers to increase their engagement and well-being. In order to experience more positive emotions, teachers might also benefit from specific interventions that might be drawn from positive psychology, such as mindfulness meditation, loving-kindness meditation (see, for review, Garland et al., 2010), interventions focused on work–family balance (Crain et al., 2017), and cultivation of positive teacher–student relationships (Cook et al., 2018). Moreover, teachers’ positive emotions and engagement could be cultivated through poetry, martial arts, and music, as these practices have been related to higher levels of flourishing and engagement (Croom, 2012, 2014, 2015).

Our findings support the importance of addressing positive emotions in programs aimed to build teachers’ engagement. Existing studies provided evidence for the effectiveness of work engagement interventions focused on personal resources (Knight et al., 2017, 2019; Van Wingerden et al., 2017). However, as organizational and national characteristics have been found to affect the effectiveness of interventions on engagement, it is important to adapt these interventions to teachers in different countries. In addition, our results emphasize the importance of interventions targeting employees’ well-being based on PERMA theory (i.e., positive emotions, engagement, relationships, meaning, and accomplishment) (Seligman, 2011).

### Strengths and Limitations

The present study has many strengths including considering in the same study both positive and negative emotions and the ratio between them, using a large sample size, and using an understudied sample of teachers from Eastern Europe. The limits of our study are related to teachers’ self-reports and cross-sectional design. Future studies should focus on longitudinal associations between teachers’ emotions, engagement, and long-term well-being, as change in emotions across time is an important predictor of psychological well-being (Houben et al., 2015). Daily-diary data would help to better understand whether teachers report higher levels of engagement and well-being on days when they experienced more positive than negative emotions. Future studies exploring daily dynamics of teachers’ emotions would also help us to examine the upward spiral of positive emotions; teachers’ engagement might predict further increases in positive emotions, which in turn would positively affect their well-being. Future studies should also focus on other specific positive emotions, such as gratitude, compassion, self-compassion, forgiveness, hope, and amusement, and negative emotions, such as boredom and anger. Despite the limitations, our study contributes to a better understanding of the mechanisms relating teachers’ emotions to work engagement and psychological well-being. Besides, our findings point out important targets for interventions designed to improve teachers’ work engagement and well-being.

## Data Availability Statement

The datasets generated for this study are available on request to the corresponding author.

## Ethics Statement

The present study was reviewed and approved by Ethical Commission of Scientific Research, University “Ştefan cel Mare” of Suceava, Romania. The participants provided their written informed consent to participate in this study.

## Author Contributions

AC planned the design of the study, organized the data collection, and drafted the methods section. Both authors developed the presented idea, and approved the submitted version. PR drafted the introduction, results, and discussion, contributed to an adequate statistical implementation of the presented idea, and computed the statistical analyses. PR contributed to the manuscript equally as AC and shares the first authorship.

## Conflict of Interest

The authors declare that the research was conducted in the absence of any commercial or financial relationships that could be construed as a potential conflict of interest.
